# Quantitative differentiation of non-invasive bladder urothelial carcinoma and inverted papilloma based on CT urography

**DOI:** 10.1186/s12894-024-01459-y

**Published:** 2024-03-26

**Authors:** Pengfei Jin, Liqin Yang, Yitao Liu, Jiehui Huang, Xu Wang

**Affiliations:** 1 Department of Radiology, Zhejiang Cancer Hospital, Hangzhou Institute of Medicine (HIM), Chinese Academy of Sciences, 1# Banshan East Road, Hangzhou, 310022 China; 2 Department of Radiology, Hangzhou Hospital of Traditional Chinese Medicine, Zhejiang Chinese Medical University, Hangzhou, China

**Keywords:** CT urography, Bladder urothelial carcinoma, Inverted papilloma of the bladder, Quantitative, Logistic regression

## Abstract

**Purpose:**

To investigate the value of CT urography (CTU) indicators in the quantitative differential diagnosis of bladder urothelial carcinoma (BUC) and inverted papilloma of the bladder (IPB).

**Material and methods:**

The clinical and preoperative CTU imaging data of continuous 103 patients with histologically confirmed BUC or IPB were retrospectively analyzed. The imaging data included 6 qualitative indicators and 7 quantitative measures. The recorded clinical information and imaging features were subjected to univariate and multivariate logistic regression analysis to find independent risk factors for BUC, and a combined multi-indicator prediction model was constructed, and the prediction model was visualized using nomogram. ROC curve analysis was used to calculate and compare the predictive efficacy of independent risk factors and nomogram.

**Results:**

Junction smoothness, maximum longitudinal diameter, tumor-wall interface and arterial reinforcement rate were independent risk factors for distinguishing BUC from IPB. The AUC of the combined model was 0.934 (sensitivity = 0.808, specificity = 0.920, accuracy = 0.835), and its diagnostic efficiency was higher than that of junction smoothness (AUC=0.667, sensitivity = 0.654, specificity = 0.680, accuracy = 0.660), maximum longitudinal diameter (AUC=0.757, sensitivity = 0.833, specificity = 0.604, accuracy = 0.786), tumor-wall interface (AUC=0.888, sensitivity = 0.755, specificity = 0.808, accuracy = 0.816) and Arterial reinforcement rate (AUC=0.786, sensitivity = 0.936, specificity = 0.640, accuracy = 0.864).

**Conclusion:**

Above qualitative and quantitative indicators based on CTU and the combination of them may be helpful to the differential diagnosis of BUC and IPB, thus better assisting in clinical decision-making.

**Key points:**

1. Bladder urothelial carcinoma (BUC) and inverted papilloma of the bladder (IPB) exhibit similar clinical symptoms and imaging presentations.

2. The diagnostic value of CT urography (CTU) in distinguishing between BUC and IPB has not been documented.

3. BUC and IPB differ in lesion size, growth pattern and blood supply.

4. The diagnostic efficiency is optimized by integrating multiple independent risk factors into the prediction model.

## Introduction

Bladder tumors are the most prevalent neoplasms of the urinary system, of which epithelial tumors accounting for over 90%, including urothelial carcinoma, low-grade malignant uroepithelial papilloma, adenocarcinoma and squamous cell carcinoma [1]. Bladder urothelial carcinoma (BUC) is the most common pathological type of bladder cancer, representing about 90% or more, which is easy to metastasize and has a high recurrence rate, leading to an unfavorable prognosis [2]. Inverted papilloma of the bladder (IPB) is a scarce benign tumor characterized by inverted growth pattern, accounting for approximately 6% of bladder tumors, with a significantly lower risk of recurrence and progression than BUC [3]. However, due to their similar clinical symptoms (such as gross or microscopic hematuria, frequent urination, dysuria), and tendency to occur in the bladder neck and triangle, distinguishing them through imaging findings can be challenging when the lesions do not invade surrounding structures, so preoperative misdiagnosis arose frequently.

The surgical approaches of BUC are classified into two categories according to the existence of muscular infiltration. Transurethral resection of the bladder tumor (TURBT) is commonly employed for non-muscle-invasive bladder cancer (NMIBC), whereas radical cystectomy is preferred for muscle-invasive bladder cancer (MIBC) [4, 5]. No matter which grade of BUC, postoperative infusion chemotherapy or systemic chemotherapy is required depending on the situation [6]. The standard surgical procedure for IPB involves TURBT, in addition to electrocauterization of bladder tumor, while preserving the bladder. Postoperative chemotherapy is not routinely administered, but regular monitoring is necessary [7]. Cystoscopy and pathological examination is the essential diagnostic tool for bladder tumors. Patients with confirmed BUC through flexible cystoscopic biopsy should undergo evaluation for myometrial invasion, often followed by re-examination using cystoscopy to extend the depth of excised tissue. If the suspicious lesion is identified as IPB by a pre-treatment imaging method before flexible cystoscopy, rigid cystoscopy and TURBT can be performed directly, and without the need for a second biopsy. In terms of prognosis, TURBT has limitations in completely removing lesions at once and often underestimates the tumor grade, resulting in a high recurrence rate of BUC. About 50%~70% of patients will redevelop tumor nodules, with most occurring within two years after surgery [8]. In contrast to BUC, IPB cells differentiating maturely, demonstrating localized growth with non-invasion of the muscular layer. The recurrence rate is pretty low, only about 1% [9]. Given the certain degree of difference in treatment options and prognosis, preoperative differentiation between BUC and IPB is quite important.

CT urography (CTU) is widely utilized in the clinical diagnosis and monitoring bladder tumor [10]. By observing pre- and post-contrast medium injection CT images, location of the tumor, number and size of lesions, relationship with surrounding tissue, as well as lymph node metastasis or distant metastasis can be accurately displayed. Studies have demonstrated that although CTU has limited value in distinguishing MIBC from NMIBC, it serves as a reference standard for staging MIBC by revealing the invasion of fat around the bladder or adjacent organs [11]. To our knowledge, there is currently no comparative study on CTU characteristics of BUC and IPB, and their role in identifying the two remains unclear.

Taken together, this study aims to analyze the differential characteristics of BUC and IPB based on CTU and to quantitatively identify them.

## Materials and methods

### Study population

This study was a retrospective study and was approved by the ethics committee of the Zhejiang Cancer Hospital. Institutional Review Board approval was obtained (202308241652000135978). Written informed consent was waived by the Institutional Review Board. The requirement for informed consent was waived by the ethics committee of the Zhejiang Cancer Hospital.

**Fig. 1:**
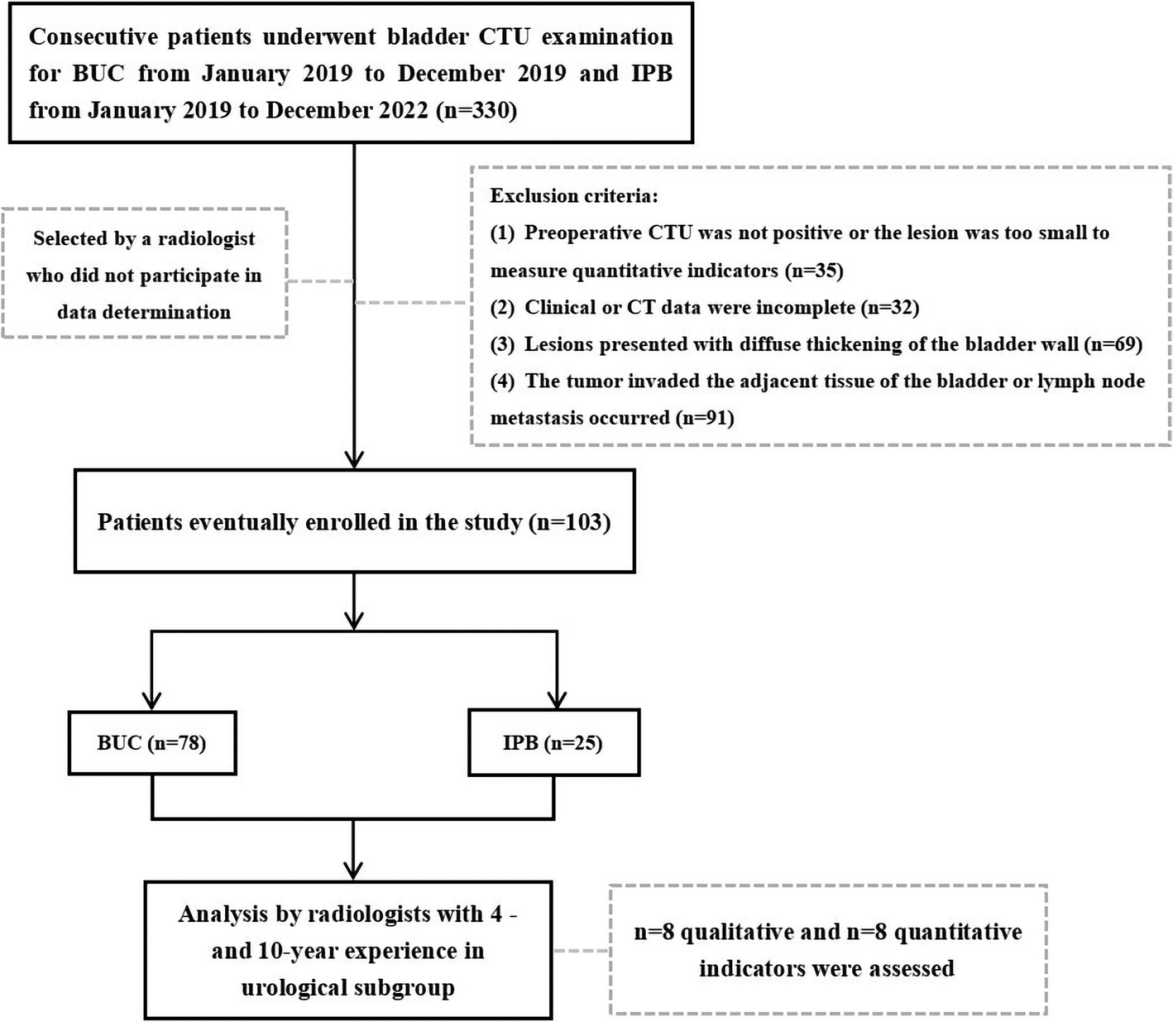
Flowchart for inclusion and exclusion of patients

We retrieved the roster of patients who underwent CTU between January 2019 and December 2022 via the PACS platform, and procured clinical (such as age, gender, urine erythrocyte) and pathological information from the electronic medical record database. The inclusion criteria comprised: (1) initial consultation for bladder lesions; (2) TURBT or radical cystectomy within 15 days after CTU examination; (3) pathologically confirmed as BUC or IPB. A total of 227 eligible patients were found. Then, some cases were further excluded according to the following criteria: (1) absence of positive CTU findings or lesions too small to measure quantitative indices; (2) incomplete clinical or imaging data; (3) lesions with diffuse bladder wall thickening; (4) peri-bladder tissue invasion or regional lymph node metastasis observed on CTU. The incidence of IPB being relatively low, we conducted a continuous collection of BUC cases from January 2019 to December 2019 and IPB cases from January 2019 to December 2022 in order to minimize the disparity in their numbers. Ultimately, 103 cases were included for successive analysis. Figure 1 shows the flow chart of this study.

### Devices and scanning methods

Image capture was performed with a special Siemens 64-layer spiral CT (Siemens SOMATOM Definition Flash, Germany). Drank 500~600ml water before examination until the patient felt the bladder was full. Patients were placed in the supine position with their foot placed anteriorly. The entire abdomen and pelvis were covered. The parameters of CT plain scan were: tube voltage 120kV, tube current was automatic with a reference value of 300mA, pitch 0.6, matrix 512 × 512, gantry rotation speed of 0.50s, collimation 128 × 0.6mm, reconstructed layer thickness 1mm, reconstructed layer spacing 1mm. Subsequently, the non-ionic contrast agent (Ultraist370, Bayer Schering, Germany) was injected intravenously with a rate of 3~3.5mL/s, and then the saline tracer 100ml was injected intravenously. The arterial, venous, and excretory phases were collected at 35s, 65s and 600~900s post-contrast injection using contrast medium tracking technique.

### Imaging interpretation

**Fig. 2:**
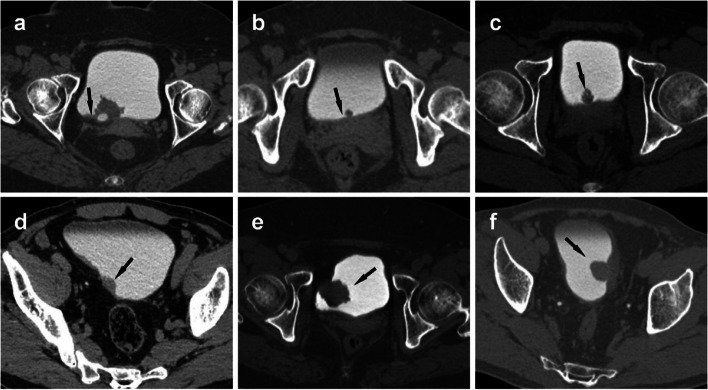
Typical images depicting junction smoothness and lesion morphology. **a** Junction smooth (arrow). **b** Junction non-smooth (arrow). **c** cauliflower. **d** hilly. **e** lobulation. **f** papillary

Four-stage CTU images of all cases were independently and randomly reviewed by two radiologists with 4-year and 10-year specializing in the urological subgroup. These radiologists were not involved in the case selection process, and clinical information as well as pathological results were concealed from them. The following specific indicators were assessed:

1. Number of lesion. A single lesion was designated as such, while the presence of two or more was considered multiple.
2. Location. According to the bladder wall where the base of the tumor was located, the growth site was divided into lateral wall, posterior wall, parietal wall, bottom wall and anterior wall.
3. Morphology. The shape of the lesion includes cauliflower, hilly, lobulation, and papillary (Fig. 2).
4. Calcification. Dense nodules exceeding 100HU on plain scan were defined as having calcification, and vice versa as not having calcification.
5. Cystic degeneration. A more hypointense area without definite enhancement within the enhanced lesion.
6. Junction smoothness. Depending on whether the bladder wall at the junction of the lesion and the bladder is elevated, thickened or rough, it was classified as smooth and non-smooth (Fig. 2).
7. Diameter measurement. The largest cross-section of the lesion was found on any orientation image during the excretion phase, the maximum transverse diameter (parallel to the base of the lesion) and the maximum longitudinal longitude (perpendicular to the base of the lesion) were measured, and the ratio of transverse to longitudinal diameter was calculated.
8. Tumor-wall interface. Defined as the length of the curved contact between the bladder wall and tumor, which was measured using the free curve caliper tool in PACS.
9. Density and enhancement. The largest level of the lesion was selected, and a 15~20mm^2^ circular ROI was placed in the solid part of the lesion with relatively uniform density in the non-enhanced and arterial phases, avoiding necrotic and calcified regions. Three consecutive measurements were averaged, and the size, plane and position of the ROI in the 2 phases were kept as consistent as possible. The arterial reinforcement rate was defined as (arterial phase CT value - non-enhanced CT value)/non-enhanced CT value.

When multiple lesions were present, the lesion with the largest area was selected as the index lesions for evaluation. The intraclass correlation coefficient (ICC) was used to evaluate the inter-rater reliability between two radiologists' measurements. If the ICC for each index exceeds 0.75, it indicated qualified inter-reader agreement, and the measurement results of 4-year experience radiologist were used for succeeding analysis.

### Construction of quantitative prediction model

Firstly, clinical characteristics including age, gender, urine erythrocyte and all quantitative and qualitative imaging features were subjected to univariate logistic regression analysis. Subsequently, variables with *P* < 0.05 were taken as related factors and multivariate logistic regression analysis was performed to determine independent risk factors for BUC and construct a combined multi-indicator model. Finally, a nomogram was created to visually display the combined model.

### Statistical analysis

The statistical analysis was conducted using SPSS 25.0 and R language 4.1.0 software. The Kolmogorov-Smirnov test was first used to check the normality of the quantitative data, and those who conformed to the normal distribution were expressed as mean ± standard deviation and compared between the two groups using the independent samples t-test; those who did not conform to the normal distribution were expressed as interquartile range (IQR) and compared between the two groups using the Mann-Whitney U test. Categorical variables were expressed as frequencies and rates, and the chi-square test or Fisher's exact test was used for comparison between two groups. The area under the curve (AUC) of the receiver operating characteristic (ROC) curve was used to evaluate the efficacy of independent risk factors and combined model to identify BUC and IPB. Sensitivity, specificity and accuracy were calculated through the Youden index. The Delong test was used to compare the differences in AUC. The nomogram underwent internal validation via 1000 bootstrap resamplings and the accuracy was assessed by Hosmer-Lemeshow test and calibration curve. A statistically significant difference was defined as *P < 0.05*.

## Results

### Baseline information of the patient

**Fig. 3:**
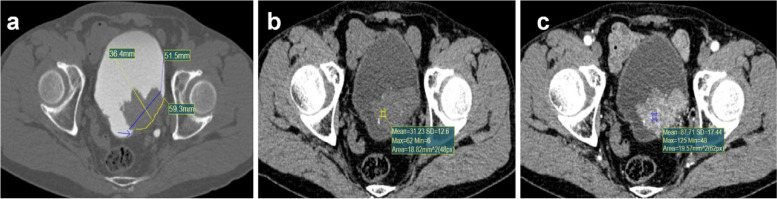
A 66-year-old male patient who underwent TURBT and was confirmed as Bladder urothelial carcinoma. **a** Axial excretory phase image showed the lesion was located at the left posterior wall, the junction between lesion edge and bladder wall was unsmooth (arrow), the maximum transverse diameter of this lesion was 5.15cm, the maximum longitudinal diameter was 3.64cm (Transverse diameter/longitudinal diameter=1.41) and the tumor-wall interface was 5.93cm (yellow curve). **b** The non-enhanced CT value of this lesion was 31.23HU. **c** The arterial phase CT value of this lesion was 87.71HU, the arterial reinforcement rate was 1.81 ([87.71-31.23]/31.23)

**Fig. 4:**
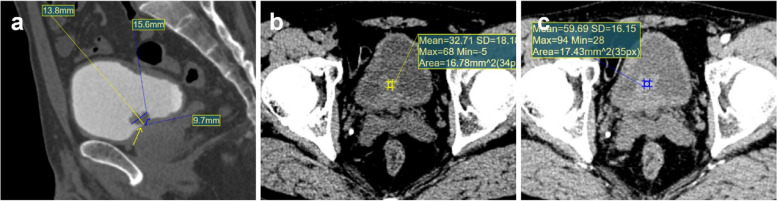
A 54-year-old male patient who underwent TURBT and was confirmed as Bladder inverted papilloma. **a** Sagittal reconstruction excretory phase image showed the lesion was located at the Bottom wall, the junction between lesion edge and bladder wall was smooth (arrow), the maximum transverse diameter of this lesion was 1.56cm, the maximum longitudinal diameter was 1.38cm (Transverse diameter/longitudinal diameter=1.13 ) and the tumor-wall interface was 0.97cm (yellow curve). **b** The non-enhanced CT value of this lesion was 32.71HU. **c** The arterial phase CT value of this lesion was 59.69HU, the arterial reinforcement rate was 0.82 ([59.69-32.71]/32.71)

Seventy-nine (76.7%) of the 103 patients were male and 24 (23.3%) were female. Based on pathological results, 78 (75.7%) BUC and 25 (24.3%) IPB were defined. Following preliminary inter-group analysis, BUC and IPB were statistically different in terms of location, morphology, junction smoothness, age and urine erythrocyte. Additionally, the maximum transverse diameter and longitudinal diameters and their ratio of BUC were larger than those of IPB (*P*≤0.001). The contact range between BUC and bladder wall was longer than that of IPB (*P*<0.001). The arterial phase CT and arterial reinforcement rate were also higher in BUC compared to IPB (*P* < 0.001). Table 1 for details. Figures 3 and 4 illustrate the characteristic CTU findings of BUC and IPB.

**Table 1:** Baseline characteristics of the study population

Characteristic	BUC(*n*=78)	IPB(*n*=25)	*P-*value
Gender, n (%)			0.471^a^
Male	58 (56.3%)	21 (20.4%)	
Female	20 (19.4%)	4 (3.9%)	
Location, n (%)			0.002^a^
Lateral wall	33 (32.0%)	1 (1.0%)	
Posterior wall	28 (27.2%)	18 (17.5%)	
Parietal wall	6 (5.8%)	1 (1.0%)	
Bottom wall	8 (7.8%)	5 (4.9%)	
Anterior wall	3 (2.9%)	0 (0.0%)	
Morphology, n (%)			0.035^b^
Cauliflower	25 (24.3%)	11 (10.7%)	
Hilly	15 (14.6%)	1 (1.0%)	
Lobulation	19 (18.4%)	2 (1.9%)	
Papillary	19 (18.4%)	11 (10.7%)	
Number of lesion, n (%)			0.089^a^
Single	57 (55.3%)	23 (22.3%)	
Multiple	21 (20.4%)	2 (1.9%)	
Calcification, n (%)			0.109^b^
Absence	64 (62.1%)	24 (23.3%)	
Present	14 (13.6%)	1 (1.0%)	
Cystic degeneration, n (%)			1.000^b^
Absence	75 (72.8%)	25 (24.3%)	
Present	3 (2.9%)	0 (0.0%)	
Junction smoothness, n (%)			0.007^a^
Smooth	27 (26.2%)	17 (16.5%)	
Non-smooth	51 (49.5%)	8 (7.8%)	
Age, mean ± SD	63.51 ± 12.23	55.00 ± 11.80	0.003^c^
Urine erythrocyte, median (IQR)	323 (6, 2647.75)	3 (0, 15)	< 0.001^d^
Maximum transverse diameter, median (IQR)	2.50 (1.80, 3.58)	1.20 (0.70, 1.70)	< 0.001^d^
Maximum longitudinal diameter, median (IQR)	1.75 (1.30, 2.40)	1.00 (0.80, 1.60)	< 0.001^d^
Transverse diameter/longitudinal diameter, median (IQR)	1.45 (1.20, 1.66)	1.00 (0.64, 1.53)	0.001^d^
Tumor-wall interface, median (IQR)	2.05 (1.30, 3.58)	0.70 (0.40, 0.90)	< 0.001^d^
Non-enhanced CT value, mean ± SD	30.95 ± 5.08	28.84 ± 5.46	0.079^c^
arterial phase CT value, mean ± SD	72.92 ± 14.88	54.49 ± 15.84	< 0.001^c^
Arterial reinforcement rate, median (IQR)	1.28 (1.07, 1.52)	0.84 (0.52, 1.19)	< 0.001^d^

*BUC* Bladder urothelial carcinoma, *IPB* Bladder inverted papilloma, *SD* Standard deviation, *IQR* Interquartile range

^a^chi-square test

^b^Fisher's exact test

^c^t-test

^d^Mann-Whitney U test

### Inter-observer agreement

The two radiologists essentially exhibited the same perception of morphology (ICC=0.777), with strong agreement on fundamental characteristics, assessment of the intersection, tumor-wall interface, diameter and density measurements (ICC range: 0.817-0.889). These results suggested that the evaluation method of this study was highly reproducible (Table 2).

**Table 2:** Inter-reader agreements of CTU features measured from the index lesion

Characteristic	ICC	95%CI
Location	0.829	0.757-0.881
Morphology	0.777	0.687-0.843
Number of lesion	0.838	0.770-0.888
Calcification	0.821	0.747-0.875
Cystic degeneration	0.853	0.791-0.898
Junction smoothness	0.841	0.773-0.889
Maximum transverse diameter	0.889	0.829-0.927
Maximum longitudinal diameter	0.877	0.823-0.915
Tumor-wall interface	0.871	0.812-0.912
Non-enhanced CT value	0.817	0.739-0.873
arterial phase CT value	0.837	0.726-0.899

*CTU* CT urography, *ICC* Intraclass correlation coefficient, *95%CI* 95% confidence interval

### Logistic regression analysis

**Fig. 5:**
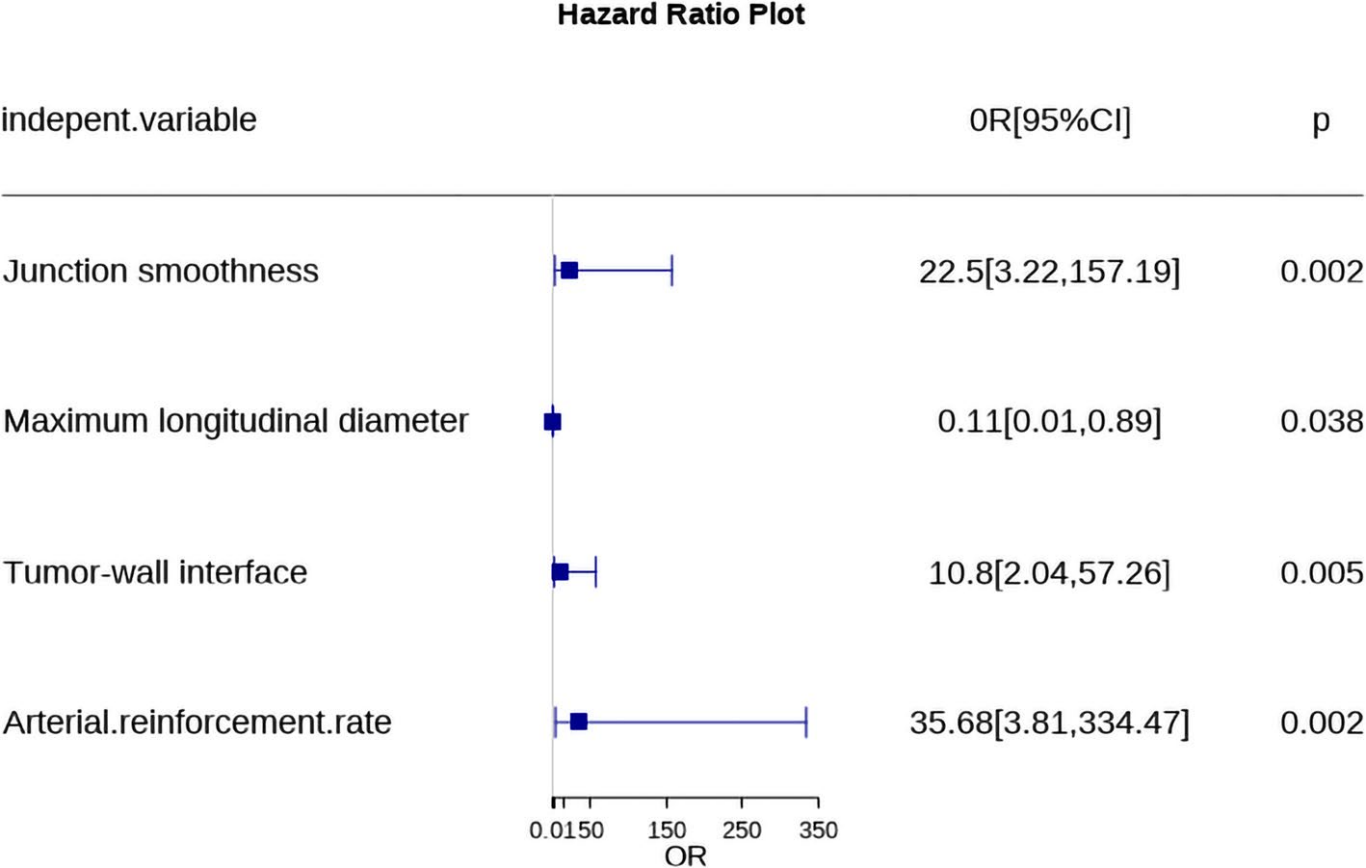
Correlation analysis of various factors between BUC and IPB

**Fig. 6:**
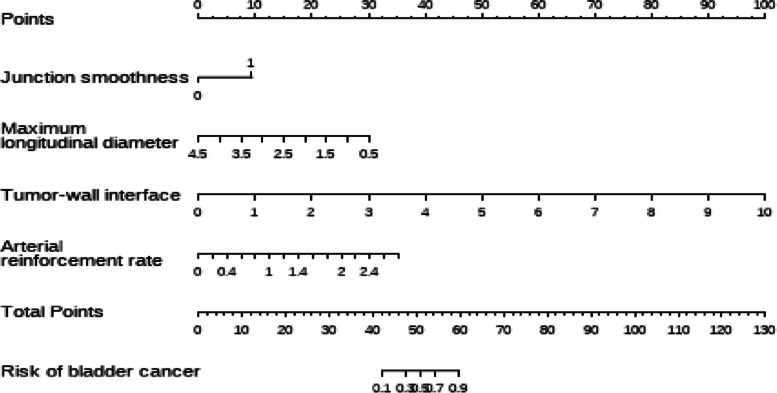
The nomogram which represents combined prediction model constructed by 4 independent risk factors

Univariate analysis showed that gender, morphology, number of lesions, calcification, cystic degeneration and non-enhanced CT value were not significantly associated with BUC (all *P* > 0.05). After excluding these indicators, the remaining features were included in multivariate logistic regression equation, which identified junction smoothness, maximum longitudinal diameter, tumor-wall interface and arterial reinforcement rate as independent risk factors for BUC (OR range 0.11- 35.68, all *P < 0.05*). Lastly, these independent risk factors were used to construct a nomogram prediction model to discriminate BUC from IPB (Table 3, Figs. 5 and 6).

**Table 3:** Univariate and multivariate analyses of clinical characteristics and CTU features

Evaluation index	Univariate logistic regression		Multivariate logistic regression	
	Odds Ratio(95%CI)	*P*-value	Odds Ratio(95%CI)	*P*-value
Gender	1.81(0.55~5.91)	0.326		
Age	1.06(1.02~1.10)	0.005	1.05(0.98~1.13)	0.156
Urine erythrocyte	1.00(1.00~1.00)	0.035	1.00(1.00~1.00)	0.154
Location				
Lateral wall	Ref.		Ref.	
Posterior wall	0.05(0.01~0.38)	0.004	0.00(0.00~3.91)	0.064
Parietal wall	0.18(0.01~3.32)	0.250	0.00(0.00~49.89)	0.129
Bottom wall	0.05(0.00~0.47)	0.009	0.00(0.00~6.97)	0.075
Anterior wall	474283.66(0.00~Inf)	0.992	5.10(0.00~Inf)	1.000
Morphology				
Cauliflower	Ref.			
Hilly	6.60(0.77~56.37)	0.085		
Lobulated	4.18(0.83~21.13)	0.084		
Papillary	0.76(0.27~2.12)	0.600		
Number of lesion	4.24(0.92~19.54)	0.064		
Calcification	5.25(0.65~42.12)	0.119		
Cystic degeneration	5217120.26(0.00~Inf)	0.991		
Junction smoothness	4.01(1.54~10.49)	0.005	22.50(3.22~157.19)	0.002
Maximum transverse diameter	3.85(1.94~7.63)	<0.001		
Maximum longitudinal diameter	4.33(1.78~10.52)	0.001	0.11(0.01~0.89)	0.038
Transverse diameter/longitudinal diameter	8.89(2.46~32.09)	0.001	0.01(0.00~21.89)	0.246
Tumor-wall interface	8.52(2.79~25.98)	<0.001	10.80(2.04~57.26)	0.005
Non-enhanced CT value	1.08(0.99~1.19)	0.082		
Arterial phase CT value	1.09(1.05~1.14)	<0.001	0.99(0.90~1.09)	0.849
Arterial reinforcement rate	24.05(4.74~122.06)	<0.001	35.68(3.81~334.47)	0.002

*95% CI* 95% confidence interval

### Diagnostic performance of independent risk factors and combined model

**Fig. 7:**
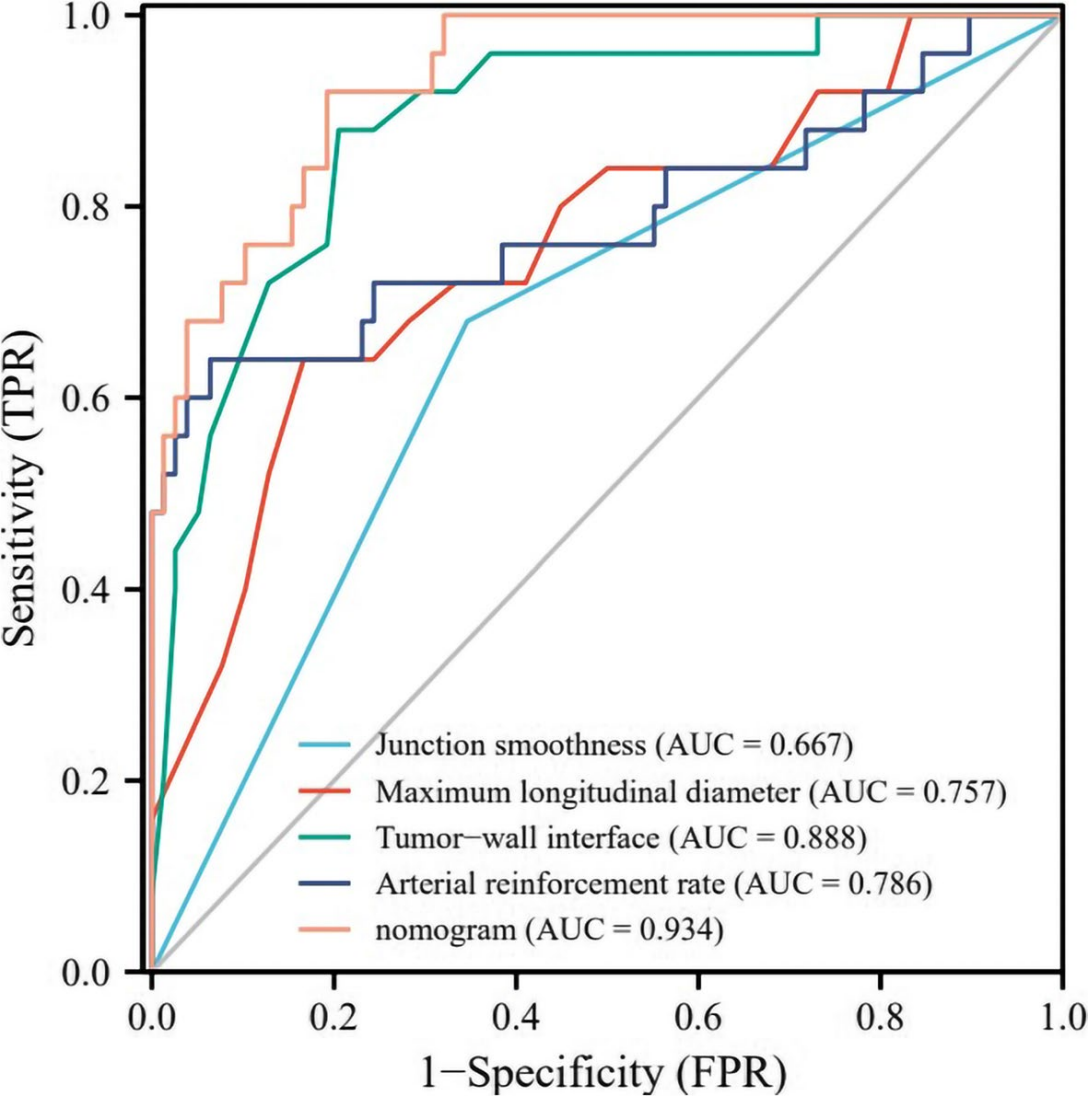
ROC curves of independent risk factors and combined model

**Fig. 8:**
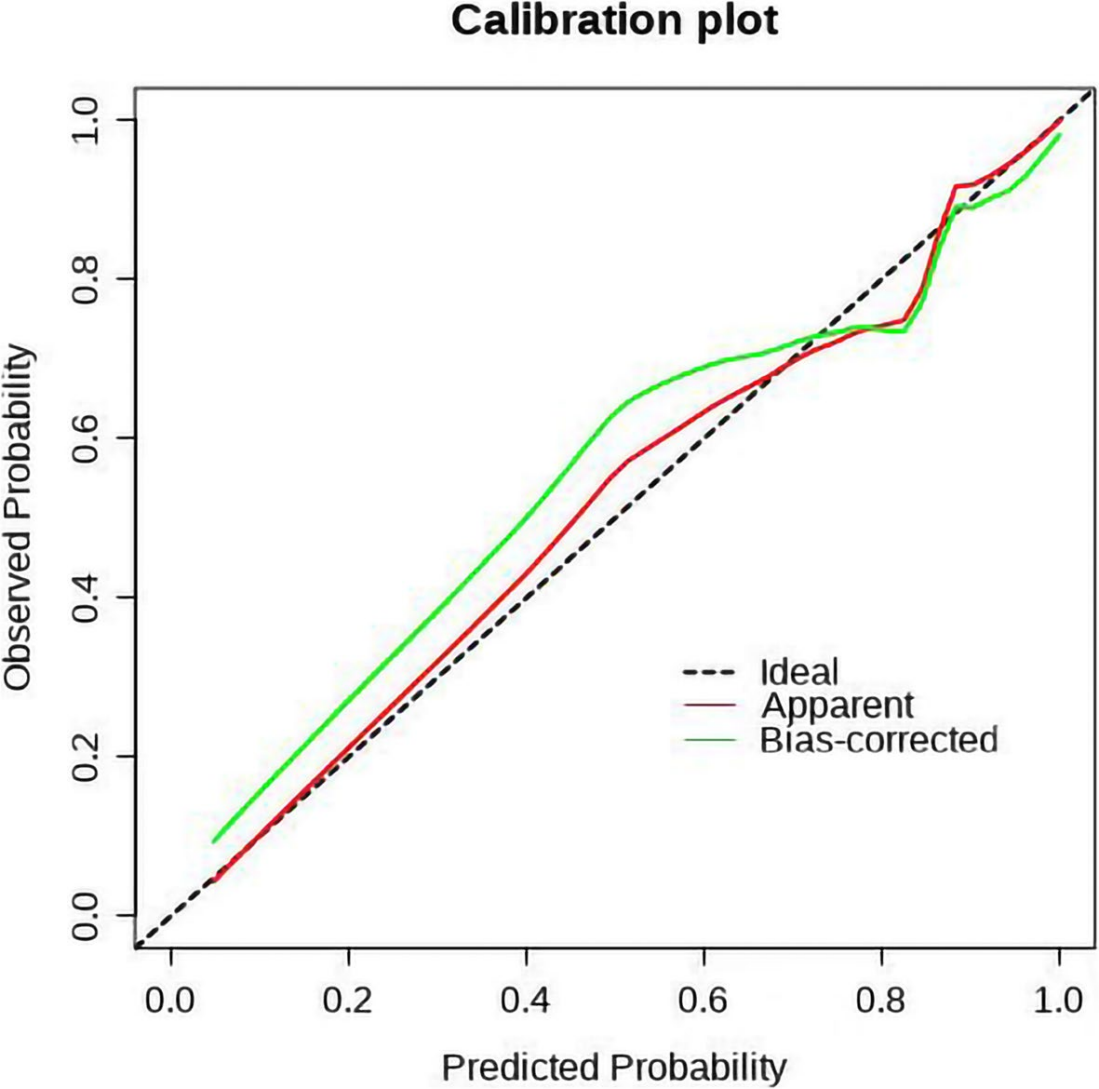
The calibration curve for predicting the urothelial carcinoma. (The Abscissa is the prediction probability, the ordinate is the actual probability, and the diagonal dashed line is the perfect prediction model. The model obtained in this study is the red line.)

According to ROC curve analysis, the diagnostic efficacy of individual independent risk factor was 0.667 (junction smoothness), 0.757 (maximum longitudinal diameter), 0.888 (tumor-wall interface) and 0.786 (arterial reinforcement rate), while the AUC of the nomogram was as high as 0.934 (95%CI: 0.887-0.981), with sensitivity, specificity and accuracy were 0.808, 0.920 and 0.835, respectively (Table 4, Fig. 7). Delong test demonstrated that the diagnostic efficiency of the nomograml was significantly higher than that of junction smoothness, maximum longitudinal diameter and arterial reinforcement rate (all *P*<0 05). Although it also surpassed the AUC of tumor-wall interface, this difference did not reach statistical significance (*P*=0.139). The Hosmer-Lemeshow test yielded x^2^=5.620, *P*=0.690, indicating that the nomogram's prediction probability did not significantly differ from that of the ideal model, thus demonstrating high accuracy. Additionally, the calibration curve demonstrated excellent agreement between predicted and actual risks for BUC occurrence (Fig. 8).

**Table 4:** Diagnostic performance of independent risk factors and combined model

Model	Cutoff	Sensitivity	Specificity	Accuracy	AUC (95%CI)	*P-*value^e^
Junction smoothness	0.500	0.654 (51/78)	0.680 (17/25)	0.660 (68/103)	0.667 (0.567~0.767)	<0.001
Maximum longitudinal diameter	1.150	0.833 (65/78)	0.640 (16/25)	0.786 (81/103)	0.757 (0.641~0.873)	0.001
Tumor-wall interface	1.050	0.795 (62/78)	0.880 (22/25)	0.816 (84/103)	0.888 (0.815~0.961)	0.139
Arterial reinforcement rate	0.943	0.936 (73/78)	0.640 (16/25)	0.864 (89/103)	0.786 (0.657~0.915)	0.010
nomogram	0.798	0.808 (63/78)	0.920 (23/25)	0.835 (86/103)	0.934 (0.887~0.981)	Ref.

*95%CI* 95% confidence interval

^e^Delong test compared with the AUC of nomogram

## Discussion

BUC is the most common malignant tumor of the bladder, characterized by strong invasiveness that facilitates infiltration into muscular layer and invade surrounding tissues. This makes complete removal the tumor via TURBT challenging, resulting in a significantly higher recurrence rate and lower survival rate for patients [12]. IPB is the most common benign tumor of the bladder, generally showing limited growth and not invading the muscular layer [13]. However, due to its unclear pathogenesis and low incidence rate, clinical symptoms and imaging manifestations often overlap with BUC, many radiologists lack sufficient experience in diagnosing it accurately unless peripheral abnormalities or signs of metastasis are observed, which is not conducive to guiding treatment and assessing patient prognosis. In this study, we examined the disparities in multiple qualitative and quantitative indicators present in CTU images between BUC and IPB, and finally searched for 4 easy-to-use quantifiable features, all of which demonstrated satisfactory diagnostic efficacy. The nomogram, constructed by the combination of 4 independent risk factors, further improved the discrimination between BUC and IPB, achieving the highest AUC and specificity. This suggests that CTU-based quantitative features may aid improve the accuracy in distinguishing between BUC and IPB preoperatively, thereby contributing to enhancing the reliability of imaging diagnostic reports.

In this study, there were no significant inter-group differences observed in terms of gender, morphology, number of lesion, calcification, cystic degeneration, or plain CT values, indicating that distinguishing BUC from IPB based solely on basic qualitative characteristics observed by the naked eye is difficult. Both of them are prevalent among men and way present with calcification, cystic degeneration, and multiple lesions are not uncommon, which appear as limited bladder wall thickening or soft tissue nodules on non-enhanced CT [14, 15]. In clinical practice, both BUC and IPB are generally considered to be prevalent in middle-aged and elderly. In all participants enrolled in this study, the mean age of BUC was higher than the that of IPB (63.51±12.23 years vs. 55.00±11.80 years, *P*=0.003), suggesting that age may serve as a potential predictive factor for differentiating them. However, the multivariate analysis failed to identify age as an independent risk factor. We postulated there were two reasons: first, the sample size was small, particularly in IPB with only 25 cases, which was insufficient to fully reflect age differences. Second, there may have been selection bias as this study included BUC that did not invade structures outside the bladder, with slower cell proliferation and smaller overall lesion size, resulting in late onset of clinical symptoms and delaying detection. However, these speculations need validation through larger-scale studies with more comprehensive staging of BUC.

Among the 4 independent risk factors, the junction smoothness was the only subjective qualitative indicator, which indirectly reflects image features around tumors. The inter-observer agreement between two radiologists' measurements was high with an ICC of 0.841. 65.4% (51/78) BUC had junction abnormalities, whereas only 32.0% (8/25) IPB caused non-smoothness at the junction. This may be attributed to their biological behavior. The tumor cells of BUC grow at different rates in all directions, resulting in irregular or lobulated tumor shapes, which promotes the precipitation and adsorption of urate crystals around the focus [16]. Moreover, about 25% of BUC infiltrates downward into the muscular layer, causing thickening and stiffening of the basal bladder wall, while the adjacent normal bladder wall was soft and mobile, creating a junction bulge or disruption of continuity [17]. IPB exhibits slow growth and regular morphology, with its surface being covered by normal urinary tract epithelium and without involving the muscle layer. There, there is no significant difference between the lesion margin and the adjacent bladder wall [18].

The maximum longitudinal diameter and tumor-wall interface quantitatively reflect the lesion size. The findings of this study showed an inverse correlation between tumor height and the likelihood of presenting with BUC. IPB typically appear as narrow basal strips or sphere-like shapes on CT images, with a longitudinal diameter similar to the transverse diameter. Previous research have shown that tipped is a specific presentation of IPB [14, 19, 20]. In contrast, the transverse diameter of BUC was significantly larger than its longitudinal diameter, indicating that the tumor show more of a broad base and the growth direction spread mainly to the bladder wall on both sides. The author posits that the aspect ratio may be a superior indicator for evaluating morphological characteristics of tumors compared to basal and height measurements, even if it was not included as a reference standard in the nomogram. For example, the aspect ratio is frequently utilized in predicting BUC staging, and one study have shown that when the aspect ratio is <0.605, it suggests a heightened likelihood of myofilament infiltration [21]. Tumor-wall interface, when used along, is the most effective independent risk factor for identifying BUC and IPB. There was no statistically different from the AUC of nomogram. This easily observable and measurable metric performed nearly perfectly in terms of inter-reader agreement. Combined use with other indicators in a multivariate logistic regression equation significantly improved the predictive efficacy, suggesting that other indicators are complementary to tumor-wall interface in predicting tumor benignity and malignancy. Previous studies have shown that the tumor-wall interface of lesions without stick was wider than that of tipped lesions, and the risk of myometrial invasion is higher [22, 23]. Ahn et al. measured the length of tumor-bladder contact on T2WI, DWI and ADC images in combination with the Vesical Imaging-Reporting and Data System (VI-RADS) to predict muscular invasion of BUC, and showed that the tumor-wall interface was an independent risk factor (OR range 1.9-2.0) for differentiating MIBC from NMIBC, compensating for the lack of VI-RADS as a qualitative indicator [24]. A variety of derived quantitative indicators have been used for staging other tumors that were similar to tumor-wall interface. For example, the extent of contact with the pleura has been used as a criterion for judging pleural invasion in lung cancer [25]. Similarly, the length of contact with the envelope has been used to predict extracapsular extension of prostate cancer [26].

Since tumor growth depends is dependent on angiogenesis, varying degrees of neovascularization and malformation can reflect different levels of tumor differentiation [27]. The presence or absence of arteriovenous fistula, vascular tortuosity, distribution, vessel size and interstitial edema can affect the perfusion of contrast medium within the lesion. The arterial reinforcement rate represents the blood supply characteristics of the lesion. The results demonstrated that the arterial reinforcement rate of BUC was significantly higher than that of IPB, indicating that the blood supply of BUC was richer than that of IPB, and the heterogeneity of tumor vessels was higher, which is consistent with the pathological manifestations [28, 29]. The hyperplastic epithelial nests of IPB are parallel arranged epithelial cells with minimal fibrovascular structures, and derive their blood supply primarily from the surrounding vasculatures of the tumor cell mass. By comparison, BUC is a malignant neoplasm characterized by extensive neovascularization and rapid growth, and most vessels extend to the interior of the tumor in the shape of dendrites at the base of the tumor. Therefore, typical BUC shows obvious enhancement in arterial phase, with maximum enhancement in venous phase and diminished enhancement in delayed phase. Conversely, the typical IPB shows moderate to severe delayed intensification. When describing the enhancement characteristics, we only analyzed the difference between arterial phase CT and non-enhanced CT value, but did not measure venous phase and delayed CT value. The reason is that the dynamic changes of the enhancement degree of lesions analyzed by simultaneous measurement of arterial and venous CT values are closely related to contrast medium injection time and individual differences such as cycle period, which may increase the error of measurement results. In addition, the contrast medium was excreted into the bladder through the urinary tract during the delayed phase, resulting in a "jet" image that interfered with accurate CT value measurement of adjacent lesions.

At present, routine examinations for the diagnosis of bladder tumors include ultrasound, CT, MRI and cystoscopy. However, due to the low soft tissue separation rate and limited scanning range of ultrasound, it is difficult to make qualitative diagnosis only according to sonogram. MRI offers clear advantages in evaluating myometrial invasion and lymph node metastasis [30], its imaging time is lengthy and image quality can be affected by intestinal peristalsis. Furthermore, the conventional scan image is thicker, and the display effect of micro-lesion and calcification is not distinct. Cystoscopy is subject to operator-dependent variability and incomplete sampling, which may lead to inaccurate pathological results. In addition, the possibility of damaging the urethra and causing urinary tract infection limits its frequent use in diagnosing and postoperative monitoring bladder tumors. In this study, CTU was used to identify BUC and IPB because of its 5 major advantages: (1) non-invasive, fast imaging and economical cost; (2) relatively objective data acquisition that is not influenced by operator level; (3) the target vessels are reconstructed using MIP mode, which can show the vascular structures within and around the lesion; (4) the excretion phase images provided clearer lesion morphology and contour details, as well as more precise diameter measurements; (5) the ability to quantitatively measure multiple features of the lesion.

The human papillomavirus (HPV) hinders the function of tumor suppressor proteins by means of its viral oncogenic proteins E6 and E7, disrupting the regulation of cell cycle and DNA repair mechanisms. Consequently, this impairs genomic stability in cells, thereby elevating the risk of cellular malignancy and tumor recurrence. The study conducted by Sarier et al. [31] involved a 2-year regular follow-up of patients with BUC, revealing that the recurrence rate was higher in HPV-positive patients (47.3%) compared to HPV-negative patients (36.8%). These findings suggest that HPV infection could potentially serve as a valuable tool for distinguishing BUC from IPB prior to surgery and assessing tumor prognosis, warranting further investigation.

Several limitations of this study are noteworthy. First, this was a single-center retrospective study, and selection bias is inevitable. Second, the sample size was small and unbalanced; therefore further studies require an increased sample size. Third, the assessment involved only a limited number of readers and did not analyze the intra-reader reproducibility of quantitative indicators. Finally, the index lesions were classified according to lesion size, which means that they may not necessarily by the highest grade lesions pathologically and could have caused some misleading results. A follow-up study will stratify the different grades of BUC and explore the differences in imaging performance with IPB.

## Conclusions

The quantitative indicators based on CTU measurement have credible consistency among readers, reflecting the variations in growth pattern, size and blood supply between BUC and IPB. In addition, the composite nomogram incorporating multiple variables may holds some benefit in helping imaging differential diagnosis of BUC and IPB, thus worth further investigation.

## Data Availability

The imaging studies and clinical data used for model development are not publicly available, because they contain private patient health information. Interested users may request access to these data, where institutional approvals along with signed data use agreements and/or material transfer agreements may be needed/negotiated. Derived result data supporting the findings of this study are available upon reasonable requests.
